# Heritability of perching behavior and its genetic relationship with incidence of floor eggs in Rhode Island Red chickens

**DOI:** 10.1186/s12711-021-00630-5

**Published:** 2021-04-21

**Authors:** Anna Wolc, Petek Settar, Janet E. Fulton, Jesus Arango, Kaylee Rowland, Danny Lubritz, Jack C. M. Dekkers

**Affiliations:** 1 Department of Animal Science, Iowa State University, 806 Stange Road, 239E Kildee Hall, Ames, IA 50010 USA; 2 Hy-Line International, 2583 240th Street, Dallas Center, IA 50063 USA

## Abstract

**Background:**

As cage-free production systems become increasingly popular, behavioral traits such as nesting behavior and temperament have become more important. The objective of this study was to estimate heritabilities for frequency of perching and proportion of floor eggs and their genetic correlation in two Rhode Island Red lines.

**Results:**

The percent of hens observed perching tended to increase and the proportion of eggs laid on the floor tended to decrease as the test progressed. This suggests the ability of hens to learn to use nests and perches. Under the bivariate repeatability model, estimates of heritability in the two lines were 0.22 ± 0.04 and 0.07 ± 0.05 for the percent of hens perching, and 0.52 ± 0.05 and 0.45 ± 0.05 for the percent of floor eggs. Estimates of the genetic correlation between perching and floor eggs were − 0.26 ± 0.14 and − 0.19 ± 0.27 for the two lines, suggesting that, genetically, there was some tendency for hens that better use perches to also use nests; but the phenotypic correlation was close to zero. Random regression models indicated the presence of a genetic component for learning ability.

**Conclusions:**

In conclusion, perching and tendency to lay floor eggs were shown to be a learned behavior, which stresses the importance of proper management and training of pullets and young hens. A significant genetic component was found, confirming the possibility to improve nesting behavior for cage-free systems through genetic selection.

## Background

With recent changes in commercial egg production systems, which are moving from cages towards floor, aviary, and free-range systems in some regions of the world, the importance of behavioral traits has increased in layers. One of the traits that is important in non-cage systems is the use of perches, which allows birds to exercise and use vertical space within the housing system. Perching not only meets a behavioral need of birds but also contributes to better muscle development and bone mineralization, better feather cover on the back, and better foot and nail health [1]. Increasing vertical space also reduces the effective bird density and allows birds to rest on an elevated perch and avoid unwanted social interactions. However, these benefits come at the cost of lower feed efficiency, greater keel damage, higher mortality and other welfare concerns [1, 2]. Recording perch use requires observation of birds in person or by camera in order to capture the behavior. We have not found estimates of genetic parameters of perching in the published literature, but strain differences were noted for perching [3] and spatial distribution in aviaries [4], which suggest that there may be a genetic component to this type of behavior.

Perch use, which involves the ability to jump, may be genetically related to other behavioral traits that are important in cage-free systems, such as the incidence of mislaid eggs. Proper use of nest boxes is critical for food safety because floor laid eggs carry a risk of microbial contamination. Moreover, floor eggs must be manually collected, which increases both labor costs and the proportion of second grade (lower value) eggs, thus negatively affecting the farm economics. Between-animal differences in nest use and in the ability to learn nest use were shown by Cooper and Appleby [5] and Settar et al. [6]. Learning ability for nest use was demonstrated by a significant decline in the proportion of floor eggs with age of hens [5]. Collection of data for floor- versus nest-laid eggs at the level of individual birds is expensive and labor intensive because it requires trap nesting, with regular checking and release of hens from the nests. With the availability of newer technologies of funnel nests [7] or SmartNests [8], it is now possible to record various nesting-related traits, including the time of day the nest is used, the total time spent in the nest, and individual nest box preferences. However, due to the high cost of such equipment, these studies can be performed only on a limited number of birds. Other technologies are being considered for identification of individual birds in group housing, including face recognition and RFID tagging. The use of traditional paint marking is not practical in brown feathered birds and would not be feasible in a study of this scale. However, recent advancements in technology allow facial recognition in chickens, as proposed by the ZhongAn company in a “GoGo chicken” project [9] or tracking movement of individuals in a group setting with RFID tags and accelerometers [10, 11]. With further development of these tools and decreasing costs, more accurate evaluation of behavior on an individual level in a group setting will become possible on a large scale. Another approach to identify layers of floor eggs in breeder flocks is to use genomic information from embryonic DNA to identify the individual hen laying on the floor.

Multiple management interventions can be employed to significantly reduce incidence of floor eggs, such as rearing on the floor with access to perches [12, 13] timing of feeding [14], restricting litter floor access [15], minimizing shaded areas, and avoiding deep litter in corners of the lay house. However, genetic components of nesting behavioral traits have also been reported [16]. Heritability of nesting behavior was estimated by Icken et al. [7] using traits recorded with the funnel nest, but the relatively small sample sizes resulted in high variability of heritability estimates between flocks and production periods (between 0 and 0.56). Using family-based pens, Settar et al. [6] estimated moderate heritabilities of the proportion of floor eggs, ranging from 0.39 to 0.44.

Against this background, the objectives of this study were to estimate the heritability of percent of birds observed perching, its genetic correlation with the incidence of floor eggs, and to determine whether there is a genetic component to learning ability. In addition, the possibility of using genomic information to identify hens that lay floor eggs was explored.

## Methods

### Housing

All birds used in this study were handled according to Hy-Line International animal welfare policy approved by the veterinarian on staff. Hens of two purebred Rhode Island Red lines (Table 1) were placed in multibird floor pens within the same testing facility, as described by Settar et al. [6]. All hens were reared in multibird wire cages without enrichment to avoid prior exposure to perches and encourage maximum expression of genetic differences in floor egg behavior after transfer to a floor pen laying house at 17 weeks of age. Both grow and lay houses were environmentally controlled, with feed provided ad libitum according to breed recommendations. In the lay house, hens were managed in multibird wooden floor pens (size 1.1 m * 1.96 m * 1.51 m, with 12 to 16 females per pen) by sire family, with a minimum of two pens per sire. Different perch options were provided in each pen, including in front of the nest boxes and over the feed line, as well as a wooden board across the back side (1.5 m) of the pen. Each pen had a nipple water system and eight metal nest boxes without nest pads organized in two levels. The number of nest and floor eggs was collected daily by pen. The number of hens perching was recorded daily during a single visit in the house during the egg collection. A single person per day was responsible for data recording within the barn but the person could vary between days, the activity of the person could have influenced the birds’ behavior thus we ensured that each sire had two replicated pens with different locations in the barn. Records were accumulated into weeks to reduce random variation, improve normality of the distribution of the data, and reduce inflation at zero for the perch data. Data from the first 11 weeks of four tests were used. Each test represented a different set of on average 97 sires (or contemporary group), with 194 pens per line. For each test, hens were produced in a single hatch and randomly assigned to pens within sire family. Pens were identified by sire but individual hens within a pen were not identifiable. Complete pedigree data was available for the sires for all generations with data plus two additional ancestral generations.

**Table 1:** Summary statistics of the percent of hens perching

LINE_TEST	N	mean	Sd	Median	min	max
L1_T1	1494	6.33	5.29	5.71	0	38.46
L1_T2	1639	6.56	6.39	4.76	0	45.24
L1_T3	1595	2.02	2.24	1.59	0	16.67
L1_T4	1326	4.92	4.20	4.44	0	40.00
L2_T1	1607	4.14	4.15	3.33	0	27.08
L2_T2	1705	4.53	4.39	3.57	0	37.50
L2_T3	1727	2.65	3.11	1.90	0	25.00
L2_T4	1403	7.49	6.23	6.67	0	34.92

### Estimation of variance components

Genetic parameters were estimated using the Average Information Residual Maximum Likelihood method, separately for each line, using the following bivariate sire model in ASReml [17]:

Model 1 $${\mathbf{y}} = {\mathbf{Xb}} + {\mathbf{Z}}_{{\mathbf{1}}} {\mathbf{a}} + {\mathbf{Z}}_{{\mathbf{2}}} {\mathbf{p}} + {\mathbf{e}},$$

where $\mathbf{y}$ is the ($\text{Nt}\times 1$) vector of weekly pen level observations or pen average across the entire test on the two traits (perching and floor eggs), $\text{N}$ is the number of weekly records, $\text{t}$ is the number of traits ($\text{t}=2$), $\mathbf{b}$ is the ($\text{Nt}\times \text{f}$) vector of fixed effects, $\text{f}$ is the number of levels for fixed effects, including test (4 levels) and the covariate of week of test nested within test (4 levels) fitted for weekly records only, $\mathbf{a}$ is the ($\text{qt}\times 1$) vector of random additive genetic effect of the sire including relationships, with $\text{q}$ the number of animals in the pedigree, $\mathbf{p}$ is the ($\text{P}\times 1$) vector of pen permanent environmental effects to account for the environmental covariance of repeated records on the pens (for pen averages permanent environment was for sire), where $\text{P}$ is the number of pens (or sires for the pen averages), $\mathbf{e}$ is the ($\text{N}\times 1$) vector of random errors, and $\mathbf{X}$ and $\mathbf{Z}$ are the incidence matrices for fixed and random effects, respectively. It was assumed that: ${\mathbf{G}} = {\text{var}}\left( {\mathbf{a}} \right) = {\mathbf{G}}_{{\mathbf{0}}} \otimes {\mathbf{A}}$, ${\mathbf{P}} = {\text{var}}\left( {\mathbf{p}} \right) = {\mathbf{P}}_{{\mathbf{0}}} \otimes {\mathbf{I}}_{{\mathbf{n}}}$, and ${\mathbf{R}} = {\text{var}}\left( {\mathbf{e}} \right) = {\mathbf{R}}_{{\mathbf{0}}} \otimes {\mathbf{I}}_{{\mathbf{n}}}$, for one record per trait and no missing data, where $\mathbf{A}$ is the $\text{q}\times \text{q}$ matrix of additive relationships between all animals in the pedigree, ${\mathbf{I}}_{\mathbf{n}}$ is the identity matrix of order $\text{n}$, ${\mathbf{G}}_{{\mathbf{0}}}$ is the $\text{t}\times \text{t}$ (co)variance matrix of additive genetic effects, ${\mathbf{P}}_{{\mathbf{0}}}$ is the $\text{t}\times \text{t}$ (co)variance matrix of pen permanent environmental effects, ${\mathbf{R}}_{{\mathbf{0}}}$ is the $\text{t}\times \text{t}$ (co)variance matrix of residuals, $\otimes$ denotes the Kronecker product, hence: ${\text{E}}\left( {\mathbf{y}} \right) = {\mathbf{Xb}}$ and ${\text{var}}\left( {\mathbf{y}} \right) = {\mathbf{ZGZ}} {\text{'}} + {\mathbf{R}}$.

The dataset was skewed for both traits but REML has been shown to be resistant to deviations from normality [18], so no data transformation was applied in order to retain easily interpretable results. Convergence was assumed when the log-likelihood changed by less than 0.002 times the number of iterations and variance estimates changed by less than 1% [17].

Heritability for trait i was estimated using the [i,i] element from the estimated matrices of variance components as $\frac{4*\text{var}(\text{a})}{4*\text{var}\left(\text{a}\right)+\text{var}\left(\text{p}\right)+\text{var}(\text{e})}$. Similarly, repeatability was estimated as $\frac{4*\text{var}\left(\text{a}\right)+\text{var}(\text{p})}{4*\text{var}\left(\text{a}\right)+\text{var}\left(\text{p}\right)+\text{var}(\text{e})}$.

Estimates from the fixed regression part of this model were used to characterize trends in the traits over the duration of the test.

To investigate genetic differences in learning, weekly records were also analyzed using the following random regression model, which was expanded to a bivariate form with the same model equation:

Model 2 $${\mathbf{y}} = {\mathbf{Xb}} + {\mathbf{Z}}_{{\mathbf{1}}} {\mathbf{a}} + {\mathbf{Z}}_{{{\mathbf{1}}_{{\mathbf{r}}} }} {\mathbf{a}}_{{\mathbf{r}}} + {\mathbf{Z}}_{{\mathbf{2}}} {\mathbf{p}} + {\mathbf{Z}}_{{{\mathbf{2}}_{{\mathbf{r}}} }} {\mathbf{p}}_{{\mathbf{r}}} + {\mathbf{e}},$$

where all terms are the same as in Model 1, except ${\mathbf{a}}_{\mathbf{r}}$ and ${\mathbf{p}}_{\mathbf{r}}$ are vectors of regression coefficients on week of random additive genetic and permanent environmental pen, respectively, and ${\mathbf{Z}}_{{{\mathbf{1}}}_{\mathbf{r}}}$ and ${\mathbf{Z}}_{{{\mathbf{2}}}_{\mathbf{r}}}$ are the corresponding matrices with covariates for week (fitted without standardization or centering). In addition, a separate residual variance was fitted for each week of test (homogenous residual variance did not resolve convergence problems but decreased the goodness-of-fit of the models). The variances and covariances of the random effects can be written as: $\text{var}(\mathbf{a},{\mathbf{a}}_{\mathbf{r}})=\mathbf{A}\otimes \mathbf{K}$, $\text{var}(\mathbf{p},{\mathbf{p}}_{\mathbf{r}})=\mathbf{P}\otimes \mathbf{K}$, ${\text{var}}\left( {\mathbf{e}} \right) = {\mathbf{R}}_{{\mathbf{0}}} \otimes {\mathbf{I}}_{{{\mathbf{ni}}}}$, where $\mathbf{K}$ is the $2\times 2$ matrix of genetic (co) variances for intercept and slope and ${\mathbf{R}}_{{\mathbf{0}}}$ is the residual covariance matrix.

Variance components for each week of tests were obtained from a matrix $\mathbf{L}\mathbf{K}\mathbf{L}{\text{'}}$ where $\mathbf{L}$ is the vector of polynomial coefficients. The genetic (co)variance matrix was obtained by multiplying elements of the sire (co)variance matrix by 4.

For single-trait analyses for percent of hens perching in Line 1, the estimate of the variance of ${\mathbf{p}}_{\mathbf{r}}$ was not different from zero and was dropped from the model. Also, ${\mathbf{a}}_{\mathbf{r}}$ had to be dropped from the bivariate model for percent of hens perching to enable convergence for Line 1. Thus, for the random regression model in Line 1 estimates of heritability are shown from both the more complex single-trait model (no ${\mathbf{p}}_{\mathbf{r}}$ but including ${\mathbf{a}}_{\mathbf{r}}$ for % perching) and the simplified bi-variate model (no ${\mathbf{p}}_{\mathbf{r}}$, no ${\mathbf{a}}_{\mathbf{r}}$). Full model converged for incidence of floor eggs in both lines and for percent perching in Line 2.

#### Potential of genomic data to identify floor eggs

To evaluate the possibility of identifying hens that lay floor eggs using genomic information, a small pilot study was designed. Floor eggs collected on one of the test days were sent to the hatchery for incubation. On day 7 of embryonic development, DNA was extracted from embryo tissues and genotyped using single-plex KASP genotyping [19] with a custom parentage set of 242 SNPs. The same SNPs were also genotyped on all males and females from the same pens. Parentage was assigned based on minimum number of parent–offspring mismatches for all possible pairs. This allowed validation of a protocol for genotyping embryo tissues and parentage assignment. To test the accuracy of parentage assignment, a single blinded experiment was run in parallel with 60 hatching eggs with pedigree known to one of the co-authors. The same process of incubation, DNA extraction and genotyping as previously described was applied. In this case parentage analysis was tested against all possible male and female breeders from that line with the parental genotypes extracted for the same 242 SNP from their previously determined Axiom50k SNP chip genotypes.

## Results and discussion

Perch use in this study (around 5% of hens observed perching at the time of visit, Table 1) was lower than a previously reported daytime perch use of 10% [20] or even higher values (> 25%) reported for example by Valkonen et al. [21] and Barnett et al. [22]. This could be related to less accurate recording (a single time point per day) vs the continuous monitoring that was used in some of the other studies and also due to the specific pen design. Optimal recording of this trait would combine continuous monitoring with individual bird identification, but that was not available for this study. The hens within this test had been reared in cages without perches. Under rearing conditions more appropriate for cage-free birds, floor pen rearing, perching, and multi-level environment, the use of perches is expected to be greater and the proportion of floor eggs is expected to be lower than in the current study. The rearing protocols used here were expected to maximize variation between families and to allow a more accurate breeding value prediction for nesting behavior.

### Trends of percent hens perching and percent floor eggs over weeks of test

**Fig. 1:**
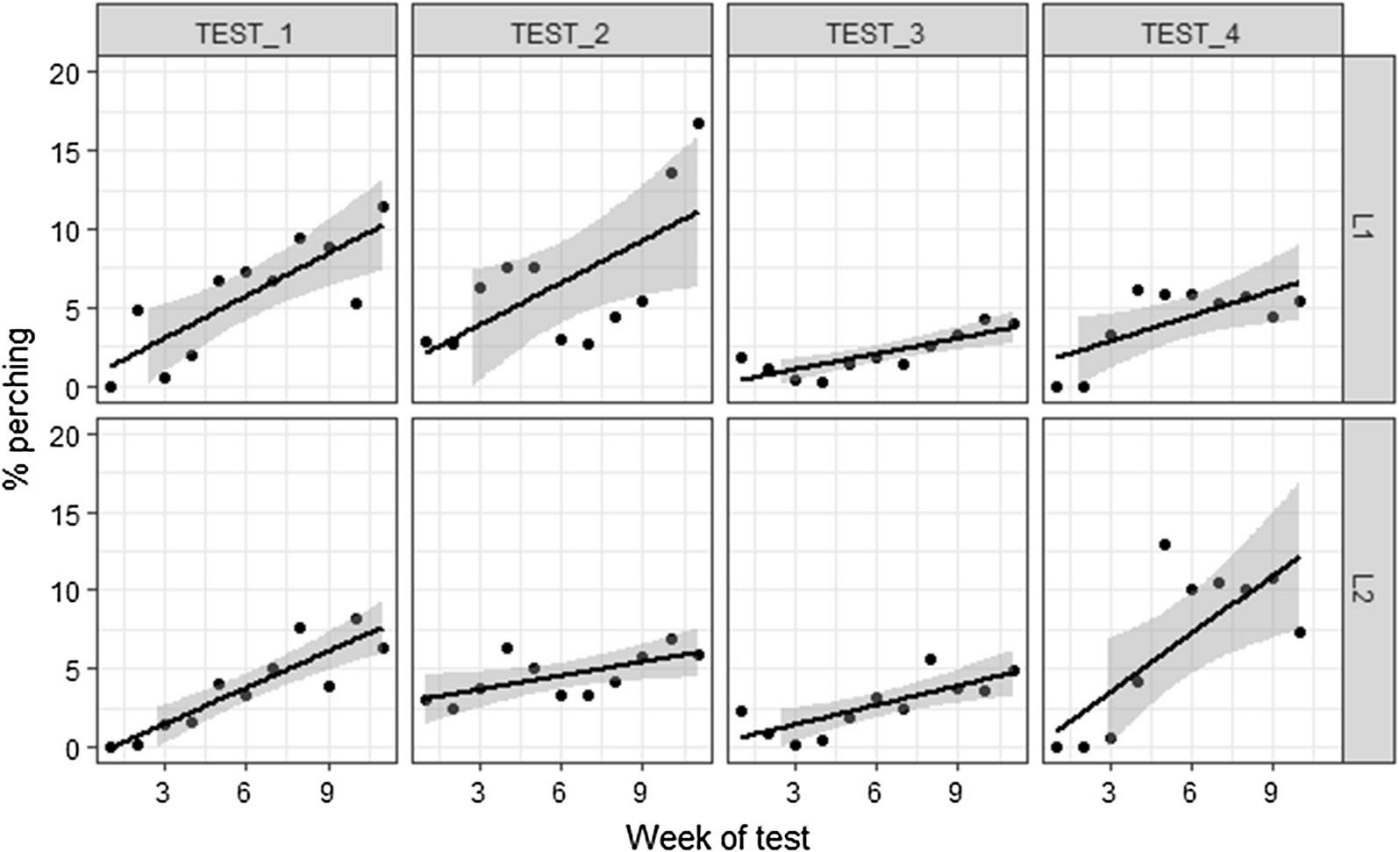
Percent of hens observed perching over four (11-week) rounds of testing in two lines. Data points represent weekly averages expressed as deviations from the percent hens perching in week one for each test by line combination. Regression lines show the trends in the means over duration of the test

Both lines showed a significant increasing trend in the proportion of perching hens over the duration of the test, with slopes varying between the tests (Fig. 1).

One of the contributing behaviors in addition to perch use per se could have been getting used to the person performing the test, thus reducing fear and a greater tendency to remain perched. Estimates of the fixed regression coefficients of % perching on weeks of test from Model 1 ranged for L1 from 0.24 in TEST_3 to 0.92 in TEST_2 and for L2 from 0.29 in TEST_2 to 1.11 in TEST_4 (all standard errors were below 0.05). This indicates that the hens learn perch use and better use vertical space with time. The tendency to increase use of perches with age was also shown in broilers [23] and young layers without prior perch experience [20]. Similar to perch use, hens from both lines showed an ability to learn to use nests, decreasing the percent of floor eggs over time, with slopes varying between tests from − 4.89 to − 3.32% per week for L1 and from − 3.53 to − 2.47% for L2. Because perch use increased and floor eggs decreased with age, a negative correlation was expected between these traits, but with lots of variation, there was only a weak (− 0.09 to − 0.05) negative phenotypic correlation between the pen-level records for these traits (Table 2), possibly due to overall low perch utilization.

**Table 2:** Estimates ± SE of genetic parameters for percent floor eggs and percent hens perching in two lines

	Weekly records		Average	
	Line 1	Line 2	Line 1	Line 2
Heritability % perching	0.22 ± 0.04	0.07 ± 0.05	0.66 ± 0.09	0.24 ± 0.16
Heritability % floor eggs	0.52 ± 0.05	0.45 ± 0.05	0.71 ± 0.08	0.66 ± 0.09
Repeatability % perching	0.33 ± 0.03	0.26 ± 0.03	0.79 ± 0.03	0.59 ± 0.07
Repeatability % floor eggs	0.71 ± 0.03	0.66 ± 0.03	0.85 ± 0.03	0.82 ± 0.03
Genetic correlation	− 0.26 ± 0.14	− 0.19 ± 0.27	− 0.06 ± 0.20	− 0.20 ± 0.36
Permanent environmental correlation	− 0.04 ± 0.07	− 0.14 ± 0.07	− 0.09 ± 0.14	− 0.15 ± 0.17
Residual correlation	0.02 ± 0.01	0.01 ± 0.01	− 0.02 ± 0.01	− 0.11 ± 0.01
Phenotypic correlation	− 0.09 ± 0.05	− 0.05 ± 0.04	− 0.08 ± 0.12	− 0.13 ± 0.12

The analysis is based on weekly records and the average across the entire testing period

### Bivariate repeatability model for weekly data and pen averages

Estimates of genetic parameters from Model 1 are summarized in Table 2. Based on weekly records, the percentage of hens observed perching had a lower heritability (~ 0.2 in Line 1 and 0.07 in Line 2) than the percentage of floor eggs (~ 0.5). The moderate heritability for percent floor eggs for both lines confirms the results obtained by Settar et al. [6] for the same breed of chicken and was within the range of estimates reported by Icken et al. [7]. Repeatability for percent floor eggs was moderate for weekly records and high for pen averages suggesting that the data collection is sufficient to capture differences between pens and families; for percent perching, repeatability was low to moderate suggesting that an additional recording might be beneficial. Estimates of the genetic correlation between the two behavior traits analyzed were negative and stronger than correlations at the phenotypic level but with large standard errors. Residual and permanent environmental correlations were close to zero for both lines. For pen averages, the heritability estimates were close to the estimates of repeatability from the weekly records, except for percent perching in Line 1. The estimate of genetic correlation was similar (around − 0.2) for weekly records and pen averages for Line 2 but was numerically stronger for weekly records in Line 1.

### Random regression model

**Fig. 2:**
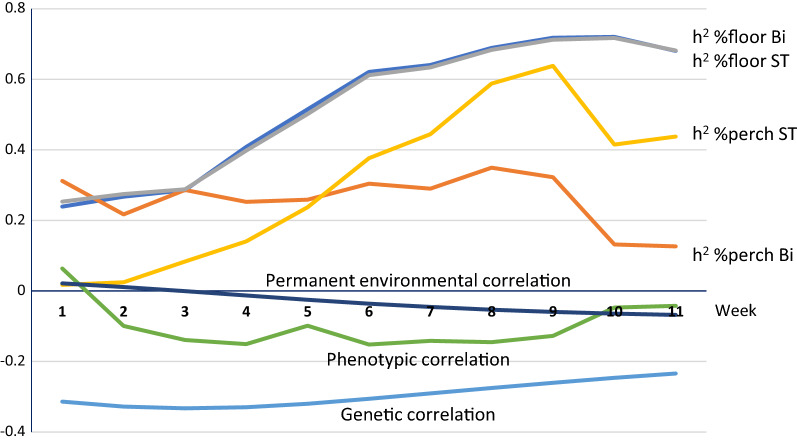
Estimates of genetic parameters for percent floor eggs and percent hens perching for Line 1. Estimates are shown across 11 weeks of test based on the single-trait (ST) and bivariate (Bi) random regression model in Line 1

**Fig. 3:**
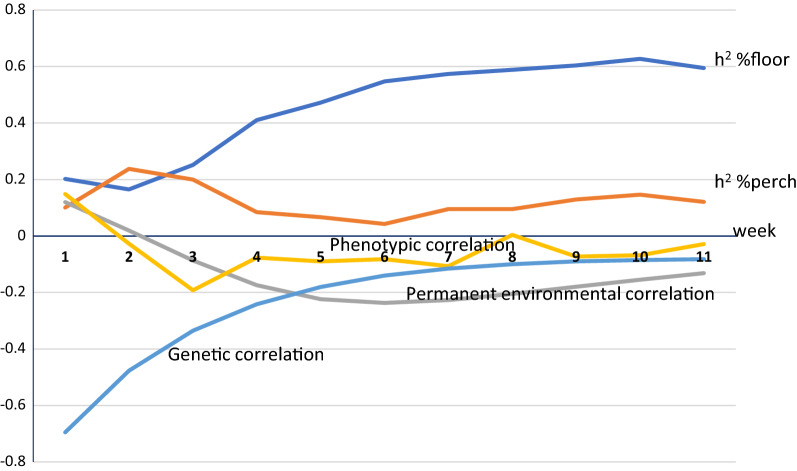
Estimates of genetic parameters for percent floor eggs and percent hens perching for Line 2. Random regression estimates of heritability for percent floor eggs and percent hens perching and genetic and phenotypic correlations between these traits across 11 weeks of test in Line 2

Results from the random regression model are shown in Figs. 2 and 3.

For both lines, heritability for percent hens perching was low at the beginning of the test (around 0 for Line 1 and around 0.2 for Line 2) but in Line 1 increased during the testing period, starting from week 4. For the final bi-variate model for Line 1, in which only the residual variance was allowed to change over the duration of the test for percent hens perching, the estimate of heritability for percent hens perching remained around 0.2, consistent with results of Model 1 (Table 1). Heritability for percent floor eggs was low at the beginning of the test (around 0.2) but increased around the 4th week of test for both lines. The estimates of genetic variance are in Table 3.

**Table 3:** Estimates ± SE of genetic variance for percent floor eggs and percent hens perching using random regression model

	Line 1		Line 2	
	Intercept	Slope	Intercept	Slope
Percent perching	0.56 ± 1.3^a^, 5.7 ± 1.3^b^	0.31 ± 0.06^a^	1.8 ± 0.7	0.06 ± 0.03
Percent floor eggs	270.5 ± 87.0^a^, 262.7 ± 85.5	7.2 ± 1.7^a^, 6.6 ± 1.6	132.8 ± 52.4	4.0 ± 1.3

^a^Single trait random regression model

^b^Bivariate model for Line 1 did not include slope

In spite of a non-significant genetic component for the intercept for perching in Line 1 across the whole period, the results from the random regression model confirmed the overall higher heritability for percent hens perching in Line 1 than in Line 2. Both lines showed non-zero genetic variance in slopes for both traits, confirming genetic differences in learning of perch usage and nest use.

Estimates of the genetic correlation between percent hens perching and percent floor eggs from the final model for Line 1 (no slope for permanent environmental effect and no intercept genetic effect for percent hens perching) were consistently below zero (around − 0.32) across the 11 weeks of the test. For Line 2, based on the full model, estimates of the genetic correlation between percent hens perching and percent floor eggs were slightly positive for the first two weeks and then negative. Estimates of the correlation for permanent environmental effects were close to zero for Line 1 but started negative and later approached zero for Line 2. Estimates of the phenotypic correlation between the two traits were close to zero across the test for both lines.

**Fig. 4:**
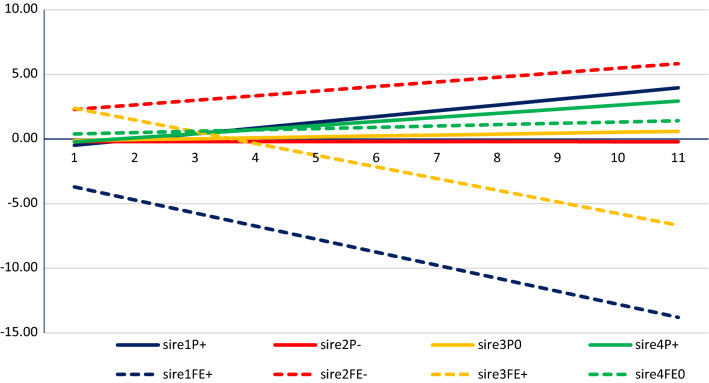
Examples of EBV for percent hens perching (P) and percent floor eggs (FE) of four sires. The four sires had daughters with different learning abilities: higher than average (+), lower than average (−) or average (0) learning ability

Figure 4 shows examples of sires with different estimated breeding values (EBV) for learning the use of perch and nest. Sire1 is the most desired for these traits, with high genetic potential for learning both perch and nest use. The opposite is true for sire2, its daughters not only starting with a higher proportion of floor eggs than the population average but also learning more slowly. Sire3 has good genetic potential for learning nest use but not for perching, while daughters of sire4 learned to use perches but showed no improvement in nesting behavior.

### Genomic analysis of floor eggs

DNA was successfully extracted and genotyped from 172 embryos from floor eggs obtained from 61 dams and 9 sires. Parentage analysis allowed a unique assignment of all parents. One of the SNPs did not show consistent calls between the KASP assay and the SNP chip and thus was excluded from the analysis. For 60 embryos of hens with known pedigree, all parents were correctly assigned based on genotypes for the 241 SNPs. We also tested what happens if the genotype of the actual dam is not available and thus ran the analysis after excluding some of the genotypes. When genotypes of some females were not available for the analysis, either parentage was not assigned or a sister of the dam was proposed as dam but with larger number of Mendelian errors than the true dam. The number of Mendelian mismatches to true parents was small (on average 0.5, maximum 3), around 10 to the sibs of true parents and on average 28 to all parental candidates, thus a smaller subset of SNPs might have been sufficient for correct parentage assignment, but the minimum required for correct results was not tested. Studies in sheep [24], goats [25] and cattle [26] suggest that as few as 100 carefully chosen SNPs would suffice for a high probability of parentage assignment. In general, the genomic approach was successful in individually identifying hens that lay floor eggs in a group setting. Some limitations include the fact that only fertile eggs can be tested. Thus, hens which did not lay fertile eggs during the collection period would be missed. There is also some level of biosecurity risk involved in incubation of floor eggs and there is significant cost and labor involved in obtaining DNA from embryos, which limits the practical implementation of this method to identify non-nest layers for selection. A minimum of two sampling periods would be recommended to address the high incidence at the beginning of the laying cycle and to identify hens that never learned to use nest later in production.

### Application

The encouraging estimates of heritability suggest that the genetic tendency to lay floor eggs can be reduced through breeding. When combined with good management, this can reduce the incidence of floor eggs in cage free systems. Possible trade-offs in terms of unfavorable genetic correlations with other welfare and/or economically important traits and loss of selection pressure on those traits will have to be further evaluated. New technologies that can help to address new challenges in changing production systems are in development, including high throughput phenotyping and genomics.

## Conclusions

Behavioral traits relevant to alternative housing systems, perching behavior and nest laying behavior, have a component of being a learned behavior, as evidenced by changes in their frequency over the testing period, demonstrating the importance of proper management of pullets and young hens for ensuring best performance of the flocks. However, both traits have been shown to have a significant genetic component in the learning ability and thus can be improved through selection in strains of hens expected to perform in alternative housing systems. Genomic methods and new technologies can be used to identify individuals expressing undesired behaviors even if housed in a group setting, thus increasing the accuracy of selection against these behaviors.

## Data Availability

The data that support the findings of this study are available from Hy-Line International but restrictions apply to the availability of these data, which were used under license for the current study, and thus are not publicly available. However, data are available from the authors upon reasonable request and with permission of Hy-Line International.
